# Mitochondrial ROS Induces Cardiac Inflammation via a Pathway through mtDNA Damage in a Pneumonia-Related Sepsis Model

**DOI:** 10.1371/journal.pone.0139416

**Published:** 2015-10-08

**Authors:** Xiao Yao, Deborah Carlson, Yuxiao Sun, Lisha Ma, Steven E. Wolf, Joseph P. Minei, Qun S. Zang

**Affiliations:** Departments of Surgery, University of Texas Southwestern Medical Center, 5323 Harry Hines Blvd, Dallas, Texas, United States of America; Wexner Medical Center at The Ohio State University, UNITED STATES

## Abstract

We have previously shown that mitochondria-targeted vitamin E (Mito-Vit-E), a mtROS specific antioxidant, improves cardiac performance and attenuates inflammation in a pneumonia-related sepsis model. In this study, we applied the same approaches to decipher the signaling pathway(s) of mtROS-dependent cardiac inflammation after sepsis. Sepsis was induced in Sprague Dawley rats by intratracheal injection of *S*. *pneumoniae*. Mito-Vit-E, vitamin E or vehicle was administered 30 minutes later. In myocardium 24 hours post-inoculation, Mito-Vit-E, but not vitamin E, significantly protected mtDNA integrity and decreased mtDNA damage. Mito-Vit-E alleviated sepsis-induced reduction in mitochondria-localized DNA repair enzymes including DNA polymerase γ, AP endonuclease, 8-oxoguanine glycosylase, and uracil-DNA glycosylase. Mito-Vit-E dramatically improved metabolism and membrane integrity in mitochondria, suppressed leakage of mtDNA into the cytoplasm, inhibited up-regulation of Toll-like receptor 9 (TLR9) pathway factors MYD88 and RAGE, and limited RAGE interaction with its ligand TFAM in septic hearts. Mito-Vit-E also deactivated NF-κB and caspase 1, reduced expression of the essential inflammasome component ASC, and decreased inflammatory cytokine IL–1β. *In vitro*, both Mito-Vit-E and TLR9 inhibitor OND-I suppressed LPS-induced up-regulation in MYD88, RAGE, ASC, active caspase 1, and IL–1β in cardiomyocytes. Since free mtDNA escaped from damaged mitochondria function as a type of DAMPs to stimulate inflammation through TLR9, these data together suggest that sepsis-induced cardiac inflammation is mediated, at least partially, through mtDNA-TLR9-RAGE. At last, Mito-Vit-E reduced the circulation of myocardial injury marker troponin-I, diminished apoptosis and amended morphology in septic hearts, suggesting that mitochondria-targeted antioxidants are a potential cardioprotective approach for sepsis.

## Introduction

Severe sepsis, associated with staggering inflammatory responses and multi-organ failure, is a leading cause of death in intensive care units [1, 2]. Despite improvements in antibiotic therapies and critical care techniques [3], there are still approximately 215,000 deaths from sepsis yearly in the United States [4]. The understanding of sepsis pathophysiology and our clinical options remain limited.

In patients with severe sepsis, the degree of mitochondrial functional deficiency associates with clinical outcomes [5, 6]. The mechanism underlying mitochondrial damage in the development of sepsis pathogenesis involves defects in energy production and induction of inflammation. A major source of mitochondrial damage comes from imbalanced production of reactive oxygen species (mtROS). MtROS subsequently impair mitochondrial structure and mitochondrial biogenesis via oxidative modifications on macromolecules, such as mitochondrial DNA (mtDNA) [7–10]. Recent investigations have also implicated mitochondria in playing a critical role in the regulation of inflammation. During cell death and organ injuries, certain molecules released from impaired mitochondria function as danger-associated molecular patterns (DAMPs) [11–14]. The list of mitochondria-derived DAMPs includes mtROS [15], mtDNA fragments [11], N-formyl peptides [16–18], ATP [19, 20], cytochrome C [21, 22], cardiolipin [23] and carbamoyl phosphate synthetase I [24].

Studies from both sepsis patients and septic animal models have indicated that a decrease in intact mtDNA content is often associated with sepsis [25, 26]. Further, circulating free mtDNA fragments have been shown to present in high quantity in both sepsis and trauma conditions [11, 27, 28]. MtDNA, similar to bacterial DNA and rich in unmethylated CpG motif, are immunostimulatory through TLR9 [11, 29, 30]. Recent investigations have shown that mitochondrial transcription factor A (TFAM), a newly identified mitochondrial DAMP [31], may directly be involved in the regulation of mtDNA-TLR9 pathway. TFAM is structurally homologous to high mobility group box protein 1 (HMGB1) [32]. HMGB1 is a known ligand for receptor of advance glycation end products (RAGE) [33]. In dendritic cells, TFAM brings RAGE together to facilitate mtDNA-TLR9-dependent cytokine production [31, 34]. However, to date, little is known about whether these signaling mechanisms are utilized in the development of multi-organ failure following the overwhelming inflammatory response in sepsis.

Cardiac dysfunction is an identified component of multi-organ failure during severe sepsis [35–37]. Septic patients with cardiac dysfunction have significantly higher mortality rates, compared to patients without this condition (70 *vs*. 20%) [38, 39]. In the heart, mitochondria comprise about 30% of myocardial volume [40]. Previously, our laboratory developed a pneumonia-related sepsis model in rats [41–45]. We have previously shown that sepsis impairs cardiac mitochondria, resulting in compromised membrane integrity, an increase in oxidative stress, and a decrease in antioxidant defense [46]. Using a mitochondria-targeting approach in this sepsis model, we have shown that mtROS-mediated mitochondria impairment clearly played a causative role in myocardial inflammation and cardiac dysfunction [47]. Our recent investigation reveals an important mechanism underling the deteriorative action of mtROS in the septic heart.

## Materials and Methods

### Ethics Statement

This study was carried out in strict accordance with the recommendations in the Guide for the Care and Use of Laboratory Animals of the National Institutes of Health. Related animal protocol and pathogen safety plan were specifically approved by Institutional Animal Care and Use Committee (IACUC) and the department of Environmental Health and Safety (EH&S) at the University of Texas Southwestern Medical Center (UTSW) (Permit Number: 2011–0073). All surgery was performed under isoflurane anesthesia and all efforts were made to minimize suffering.

### Pneumonia-related sepsis in rats

Adult Sprague Dawley rats (Charles River laboratories, Massachusetts, MA), male, 320–350 g, were used in the study. Animals were conditioned in-house for 5–6 days after arrival with commercial rat chow and tap water available at will. Sepsis was induced by intratracheal injection of *S*. *pneumoniae* type 3 (ATCC, Rockville, MD, catalog number 6303), 4x10^6^ colony forming units (CFU) per rat [46–48]. Briefly, animals were anesthetized with isoflurane and placed in a supine position. The area over the trachea was prepared with 10% povidine-iodine solution. A midline cervical incision was made, and the trachea was identified and isolated via blunt dissection. A 0.4-ml aliquot of either sterile endotoxin-free PBS or bacterial suspension was injected directly into the trachea. After the wound was closed with surgical staples, the animals were placed on a 30° incline to ensure accumulation of the injected fluid into the lungs. Our previous studies proved that the surgical procedure alone produces no ill effects.

As previously described [47], we synthesized Mito-Vit-E to >90% purity according to a published method [49]. When animals were treated, 21.5 μmoles/kg Mito-Vit-E, vitamin E or vehicle was delivered by oral gavage 30 minutes post-inoculation.

### Culture of neonatal cardiomyocytes

Neonatal rat ventricular myocytes were isolated from the ventricles of Sprague-Dawley rat pups, 2-day-old neonates (primary cardiomyocytes isolation kit, Pierce Biotechnology, Rockford, IL, catalog number 88281). Cells were pre-plated on Falcon primaria-treated tissue culture dishes at 37°C for 2 hours in order to remove fibroblasts, and then plated at a density of 1,200 cells/mm^2^ and cultured for 24 hours in DMEM/M199 (3:1) containing 5% heat-inactivated FBS, 10% normal horse serum and 1% Pen/Strep. In some experiments as detailed in figure legends, cells were treated with LPS (100 ng/ml) (Sigma-Aldrich, St. Louis, MO, catalog number L3012), Mito-Vit-E (1μM), or ODN-I (0.5 μM) (InvivoGen, Sandiego, CA, catalog number ODN 2088) 4 hours prior to harvesting.

### Preparation of tissue/cell lysates and cellular fractions

Tissues were harvested immediately following animal sacrifice, washed in PBS, snap clamp frozen, and stored at -80°C. Tissue lysates or total lysates from primary cardiomyocytes were prepared using T-PER tissue protein extraction reagent, and mitochondrial and cytosolic fractions were separated by differential centrifugation using mitochondria isolation kit for tissue (Thermo Fisher Scientific, Rockford, IL, catalog number 78510 and MITOISO1-1KT). Nuclei fractions were isolated using CellLytic Nuclear Extraction Kit (Sigma-Aldrich, Saint Louis, MO, catalog number NXTRACT-1KT). Protein concentrations were quantified by protein assay kit (BioRad, Hercules, CA, catalog number 500–0122).

### Quantification of mtDNA integrity by long PCR (LPCR)

Analysis of mtDNA integrity was performed according to a published method [50]. Briefly, tissue genomic DNA was isolated using DNeasy kit (Qiagen, Valencia, CA, catalog number 69504), digested with restriction enzyme SacII (NEB, Boston, MA, catalog number R0157S) to linearize mitochondrial DNA, and was then purified and quantified by NanoDrop ND–1000 spectrophotometer. The primers for the amplification of 14.3-kb mitochondrial genomes of both rat and mouse were 5'-ATATTTATCACTGCTGAGTCCCGTGG–3' (forward) and 5'-AATTTCGGTTGGGGTGACCTCGGAG–3' (reverse). The PCR reaction contained 0.4 ng total DNA, 1 μM of each primer, 400 μM dNTP mixtures, and 0.75 U of LA Taq enzyme (Clontech Laboratories/Takara Bio USA, Madison, WI, catalog number RR013A) in a total volume of 25 μL. The same amount (0.4 ng) of total DNA from mouse was added to serve as an internal standard. The conditions for PCR consisted of denaturation for 1 minute at 94°C followed by 25 cycles of denaturation at 94°C for 15 seconds, annealing and extension at 68°C for 10 minutes, and a final extension at 72°C for 10 minutes. The PCR products were digested with NcoI and fractionated through a 1% agarose gel. The intensities of the bands were quantified by densitometry analysis. The relative content of rat mtDNA was derived by normalization with the mouse mtDNA in each sample.

### Analysis of mtDNA oxidative damage

DNA was isolated from mitochondrial fractions using DNeasy kit (Qiagen, Valencia, CA, catalog number 69504). Levels of apurinic/apyrimidinic (AP) sites on mtDNA were determined using the DNA damage quantification kit (BioVision, Mountain View, CA, catalog number K253-25). For each measurement, 0.5 μg DNA was labeled with biotin-tagged aldehyde reactive probe via incubation at 37°C for 1 hour. The DNA was then purified, presence of AP sites was detected spectrophotometrically at OD 650 nm, and concentrations were calculated according to an avidin-biotin standard curve. Levels of 8-hydroxy-2-deoxy guanosine of mtDNA were measured using 8-OH-dG EIA assay kit (StressMarq, Victoria, BC Canada, catalog number SKT-120-96). 5 μg DNA per sample was pre-digested by nuclease P1 (1.25 U/ml in 0.625 mM ZnCl_2_ and 16.67 mM sodium acetate, pH 5.5) at 45°C for 2 hours and subsequently treated by alkaline phosphatase (1 unit per 100 μg DNA, pH 7.5–8.5) at 37°C for 1 hour. The DNA samples were then boiled and added together with an 8-OH-dG tracer, 8-OH-dG-acetylcholinesterase (AChE) conjugate, to anti-mouse IgG-coated plate. Following incubation with a mouse monoclonal 8-OH-dG antibody, substrates to AChE were added and the production of this enzyme reaction was recorded spectrophotometrically at OD412 nm. The amount of 8-OH-dG in each DNA sample was calculated according to a standard curve. All measurements were obtained in triplicate, and all other chemical reagents were from Sigma-Aldrich, St. Louis, MO.

### Assessment of Mitochondrial Outer Membrane Damage

As previously described [46, 51, 52], mitochondrial outer membrane integrity was evaluated by the measurement of cytochrome C oxidase (COX) activity in mitochondrial fractions in the presence and absence of detergent n-dodecyl β-D-maltoside (Sigma-Aldrich, Saint Louis, MO, Catalog Number CYTOCOX1). According to manufacture’s protocol, 20 μg freshly isolated mitochondrial fraction was used for each reaction. COX activity was measured by its oxidation of substrate ferrocytochrome C. Mitochondrial outer membrane damage was assessed from the ratio between the activity with and without n-dodecyl β-D-maltoside present (1 μM) under each experimental condition [53]. All measurements were performed in triplicate.

### Mitochondrial respiratory complex I-V enzyme assays

Activities of mitochondrial complex I-V were measured using enzyme assay kits according to manufacture’s protocols (Abcam, Cambridge, MA, catalog numbers ab109721, ab109905, ab109911 and ab109714). Freshly isolated mitochondrial pellets were resuspened in PBS supplemented with 10% detergent provided in the kits. Protein concentrations of these mitochondrial lysates were estimated and 25 μg (for complex I, IV and V) or 100 μg (for complex II+III) mitochondrial protein was used per reaction. Enzyme activities were measured spectrophotometricly in triplicate and expressed as changes of absorbance per minute per mg protein.

### Detection of cytosolic or extracellular mtDNA fragments

In the cytosolic fractions prepared from the heart tissue, cell pellets or culture medium from the primary cardiomyocytes, total DNA was isolated using a DNA extraction kit (Qiagen, Valencia, CA, catalog number 69506). TaqMan exogenous internal positive control (IPC) DNA (Life Technologies, Carlsbad, CA, catalog number 4308323) was spiked into all samples prior to DNA isolation as a positive control. TaqMan real time PCR (RPCR) assays were performed using primers against rat mtDNA gene NADH or IPC according to manufacture’s protocol. Samples were tested in triplicates. Results were expressed as a ratio of target gene to IPC (All RPCR reagents and gene assays were from Life Technologies, Carlsbad, CA).

### Detection of mitochondrial superoxide in cardiomyocytes

Cardiomyocytes were loaded with MitoSox Red (Invitrogen, Carlsbad, CA, catalog number M36008), a florescent dye that is specific for the detection of mitochondrial superoxide [54] (5 μM, 20 minutes, 37°C) followed by analysis with flow cytometry.

### Evaluation of mitochondrial biogenesis in cardiomyocytes

Cultured cardiomyocytes were seeded on 96-well plates and applied to the measurements of MitoBiogenesis In-Cell ELISA (Abcam, Cambridge, MA, catalog numbers ab110217) according to vender’s protocol. In this assay, the activities of two mitochondrial enzymes were measured simultaneously by spectrophotometry. The subunit I of mitochondrial complex IV (COX-I) is encoded by mtDNA whereas the 70kDa subunit of complex II (SDH-A) is by nuclear DNA (nDNA). Thus, the ratio of COX–1 activity/SDH-A activity represents the status of mitochondrial biogenesis. All measurements were obtained in triplicate and normalized by cell numbers.

### Histology analysis

#### Electron microscopy

Hearts were retrograde perfused (buffer: 4% paraformaldehyde/1% glutaraldehyde/0.1M Na Cacodylate, pH7.4). Small blocks of tissue from the mid-section of the left ventricular wall were fixed (buffer: 2.5% glutaraldehyde/0.1M Na Cacodylate, pH7.4) and examined by electron microscopy.

In addition, fresh heart tissues were fixed in 4% paraformaldehyde, transferred to PBS, and embeded in paraffin. Samples were sectioned at 5–7 μm, processed, and examined by the following approaches.

### Haematoxylin and eosin stain (H&E stain)

Tissue sections were deparaffinized, rehydrated, and incubated with hematoxylin histological staining reagent (Dako, Carpinteria, CA, catalog number S3302) for 4 minutes followed by eosin secondary counter-stain solution (Leica Microsystems, Buffalo Grove, IL, catalog number 3801600) for 1 min. Slides were then cleared by xylene, mounted with mounting medium (Thermo Fisher Scientific, Rockford, IL, catalog number 4111), and examined under Nikon Eclipse Ti-E inverted microscope at 40x magnification.

### Immunostaining of RAGE and ASC

After dewaxing and dehydration, tissue sections were blocked with 3.5% goat serum in PBS and stained with a rabbit polyclonal antibody against RAGE (Abcam, Cambridge, MA, catalog number ab3611) or ACS (Novus Biologicals, Littleton, CO, catalog number NBP1-78977) overnight at 4°C, followed by a 1-hour incubation with biotinylated goat anti-rabbit-IgG (Abcam, Cambridge, MA, catalog number ab64256) at room temperature. Staining was revealed by streptavidin peroxidase, developed with diaminobenzidine and counterstained with haematoxylin. Staining with PBS in place of primary antibody was used as negative controls. The slides were then examined under Nikon Eclipse Ti-E inverted microscope at 40x magnification.

### Terminal deoxynucleotidyl transferase (TdT)-mediated dUTP nick-end labeling (TUNEL) assay

Cardiac apoptosis was assessed by fluorometric TUNEL system (Promega, Madison, WI, catalog number G3250). According to manufacture’s protocol, tissue sections or cells were fixed and labeled with TdT reaction mixture for 1 hr at 37°C. After the reactions were terminated, the slides were sealed with and then examined under Nikon Eclipse Ti-E inverted microscope at 20x magnification. Positive nucleus staining was obtained by propidium iodide (PI) in tissue sections and by DAPI in cultured cardiomyocytes.

### Western blots

Prepared SDS-PAGE protein samples were subjected to 15% SDS-PAGE gels, and transferred to PVDF membranes. Membranes were blocked with 5% nonfat milk-PBS at room temperature for 1 hour and subsequently probed with one of the following antibodies according to experiments: GAPDH (Millipore, Billerica, MA, catalog number MAB374), c-Jun (Millipore, Billerica, MA, catalog number 09–754), NF-κB p65 (Cell Signaling, Danvers, MA, catalog number 3033S), ANT, POLG, TLR9, IRAK4, MYD88, TFAM, and caspase 1 (Santa Cruz Biotechnology, Santa Cruz, CA, catalog number sc–9299, sc–5933, sc–25468, sc–34470, sc–11356, sc–23588, sc–56036), AP-endonuclease, OGG1, UNG1(Sigma-Aldrich, St. Louis, MO, catalog number A2105, SAB2500714, PRS3865), RAGE (Abcam, Cambridge, MA, catalog number ab3611), or ACS (Novus Biologicals, Littleton, CO, catalog number NBP1-78977). The membranes were then rinsed and incubated with corresponding horseradish peroxidase-conjugated secondary antibodies (Bio-Rad, Hercules, CA, catalog number 170–6515 and 170–6516). Antibody dilutions and incubation time were according to manufacture’s instructions. Membranes were then rinsed and bound antibodies were detected by using SuperSignal West Pico Chemiluminescent Substrate (Thermo Scientific, Rockford, IL, catalog number 34077).

### Immunoprecipitation

Each 0.5 mg tissue lysate sample was pre-cleaned with 20 μl A/G plus agarose (Santa Cruz Biotechnology, Santa Cruz, CA, catalog number sc–2003) and precipitated by 20μl anti-TFAM (Santa Cruz Biotechnology, Santa Cruz, CA, catalog number sc–23588) at 4°C overnight. Following further precipitation with 20μl A/G plus agarose for 2 hours, sample was washed three times using cold PBS, solubilized with 40μl 2X SDS sample buffer (Sigma-Aldrich, Saint Louis, MO, catalog number S3401-1VL) and analyzed by Western blot.

### Measurements of IL–1β and troponin levels by enzyme-linked immunosorbent assay (ELISA)

Levels of IL–1β in heart tissue lysates and in cardiomyocytes cell lysates were examined using an ELISA kit (Biosource, Camarillo, CA, catalog number MBS162519) and results were normalized by protein amount per sample. Serum troponin-I levels were also determined by a commercial ELISA kit (Life Diagnostics, West Chester, PA) and results were normalized by serum volume. All samples were tested at least in triplicate.

### Statistical analysis

All data were expressed as mean ± SEM of at least 3 independent experiments using 6–8 animals/group. Student t-tests were used to assess the difference between two groups, such as sepsis versus sham groups and groups with drug treatments versus control groups. Bonferroni correction was applied to adjust for multiple comparisons.

## Results

### MtROS induce mtDNA damage in the heart after sepsis

In our previous study using a rat pneumonia-related sepsis model, targeted inhibition of mtROS by mitochondria-targeted vitamin E (Mito-Vit-E) improved total antioxidant capacity and suppressed H_2_O_2_ generation in cardiac mitochondria, whereas vitamin E offered little effects at the same dose [47]. In the experiments described herein, we compared the effect of Mito-Vit-E and vitamin E on cardiac mtDNA in response to sepsis.

**Fig 1:**
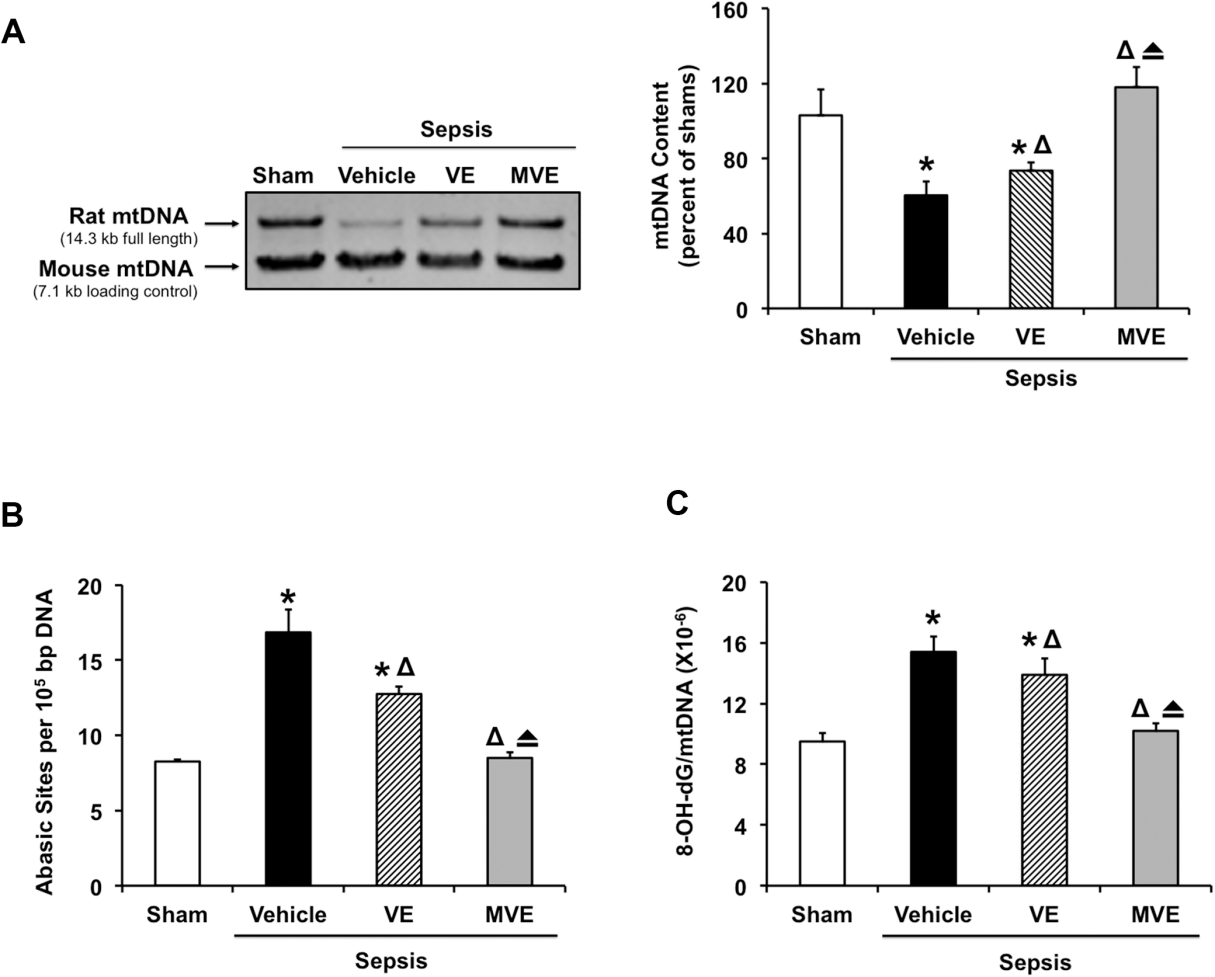
Mitochondrial ROS dependent mtDNA damage in the heart after sepsis. Rats were infected by *S*. *pneumoniae* or given PBS sham control. 21.5 μmoles/kg Mito-Vit-E (MVE), vitamin E (VE) or vehicle was administered orally 30 minutes post-inoculation, and heart tissues were harvested 24 hours later. **A.** 0.4 ng genomic DNA from the heart tissue was amplified for 25 cycles by LPCR. Mouse DNA was included as an internal control. The PCR products were digested, separated on 1% agarose gel, and analyzed by densitometry. Levels of 8-hydroxy-2-deoxy guanosine (8-OH-dG) (**B**) and apurinic/apyrimidinic (AP) sites (**C**) were measured in DNA isolated from mitochondrial fractions. All values are means ±SE. Significant differences are shown as * between sham and sepsis, Δ between vehicle and drug-treated, and **⏏** between vitamin E and Mito-Vit-E (*p*<0.01, n = 6).

First, we evaluated mtDNA integrity in the heart tissues of sham and septic rats receiving Mito-Vit-E, vitamin E, or control vehicle. Quantification of intact mtDNA content out of total genomic DNA was achieved by a long distance PCR (LPCR) method, in which the ~14kb mitochondrial genome was amplified using mtDNA specific primers [50]. LPCR condition was optimized; and a linear pattern was obtained when 0.4 ng DNA was amplified from 22 to 30 LPCR cycles (data not shown). In addition, mouse DNA was included in the LPCR reactions and utilized as an internal control, taking the advantage of the following two facts: (1) both rat and mouse mtDNA can be amplified by the same pair of primers due to sequence homology, and (2) their PCR products can be distinguished by restriction enzyme digestion. As shown in Fig 1A, genomic DNA samples prepared from the rat hearts, each included the same amount of mouse DNA as a loading control, were applied to LPCR, and the products were digested by *NcoI* and separated by electrophoresis. The upper band, 14.3 kb, represented the intact rat mtDNA, whereas the lower band, 7.1 kb, showed *NcoI*-digested mouse mtDNA. Compared with shams, septic animals showed a dramatic, ~40% decrease in intact mtDNA content. This damage in mtDNA integrity was alleviated in Mito-Vit-E treated rats, whereas vitamin E provided a slight yet significant protection.

We next examined whether Mito-Vit-E and vitamin E protect cardiac mtDNA against damage modifications after sepsis. We measured levels of 8-OHdG sites, a measure of common DNA oxidation lesions, and apurinic/apyrimidinic (AP) sites, a measure of predominant DNA damage lesions, in the mtDNA samples isolated from the heart tissues. As shown in Fig 1B and 1C, we detected a significant, over 50% increase in the generation of both 8-OHdG and AP sites on mtDNA isolated from septic animals, and an effective rescue was obtained by Mito-Vit-E but not vitamin E.

Mito-Vit-E is directly targeted against mtROS whereas vitamin E is a globally acting antioxidant, the effectiveness of Mito-Vit-E in mtDNA protection shown in these results indicates that mtROS play a significant role in causing mtDNA damage and impairment of mitochondrial genome integrity in the heart after sepsis.

### MtROS mediate a reduction in mitochondria-located mtDNA repair enzymes in the heart after sepsis

**Fig 2:**
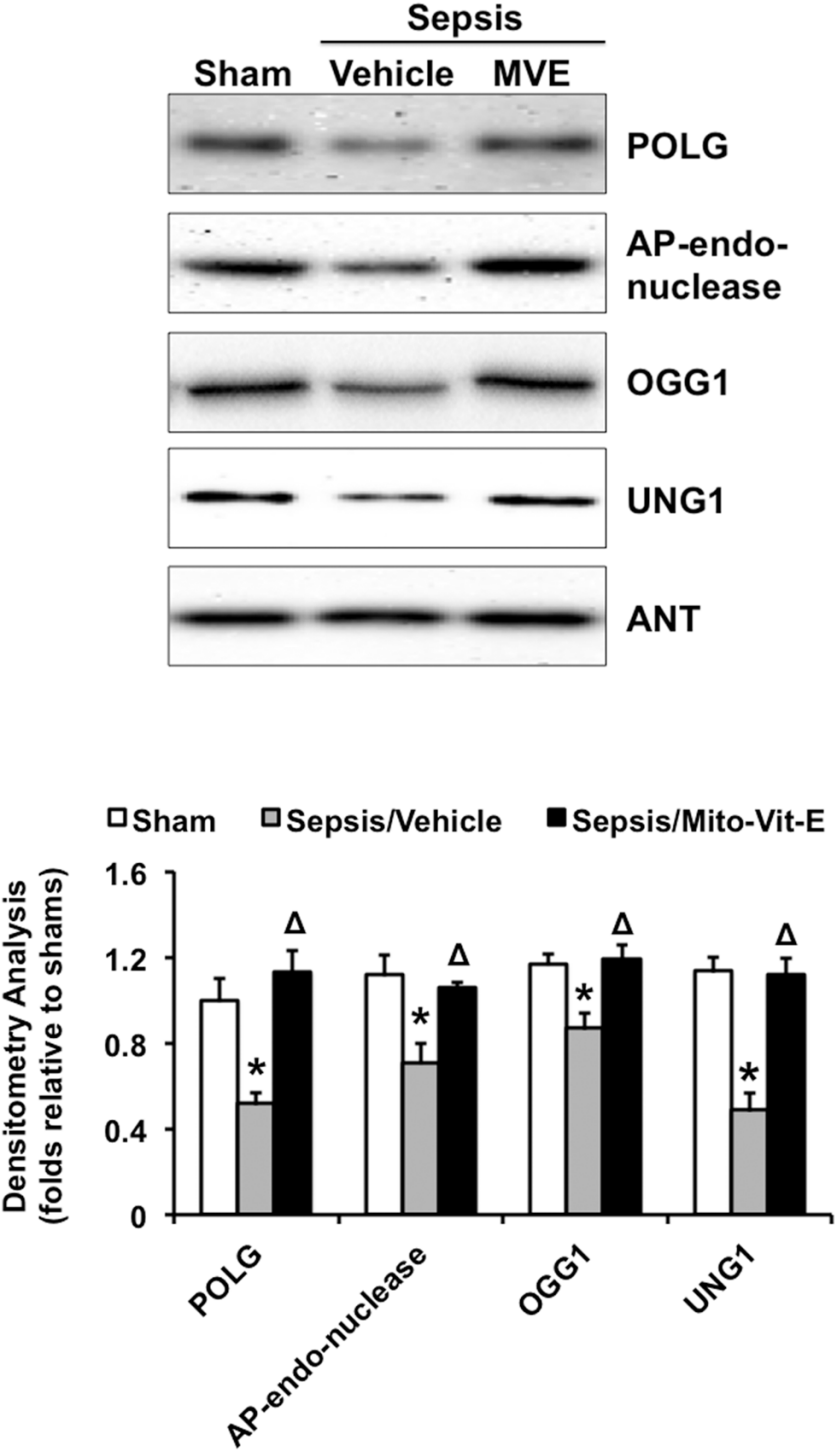
DNA polymerase γ (POLG), AP endonuclease, 8-oxoguanine glycosylase (OGG1), and uracil-DNA glycosylase (UNG1) in mitochondria. Rats were infected by *S*. *pneumoniae* or given PBS sham control. 21.5 μmoles/kg Mito-Vit-E (MVE) or vehicle was administered orally 30 minutes post-inoculation, and heart tissues were harvested 24 hours later. POLG, AP endonuclease, OGG1 and UNG1 were determined in mitochondrial fractions by Western blot using adenine nucleotide translocase (ANT) as a loading control. Results were quantified by densitometry and expressed as fold changes relative to shams. All values are means ±SE. Significant differences are shown as * between sham and sepsis and Δ between vehicle and Mito-Vit-E (*p*<0.02, n = 6).

We next examined whether mtROS damage mtDNA integrity via a down-regulation in mitochondria-localized enzymes that govern mtDNA integrity, such as mitochondria-specific DNA polymerase γ (POLG)[55] and mtDNA base excision repair enzymes including apurinic/apyrimidinic (AP)-endonuclease, 8-oxoguanine glycosylase (OGG1) and uracil-DNA glycosylase (UNG1) [56], in the heart after sepsis. We compared the levels of these enzymes in the mitochondrial fractions isolated from the heart tissue of sham and septic rats receiving Mito-Vit-E or control vehicle (Fig 2). The Western blot analysis showed that sepsis reduced their contents about 48% in POLG, 26% in AP-endoneclease, 11% in OGG1 and 25% in UNG1. Mito-Vit-E provided inhibitory effect, suggesting that sepsis-induced changes in mitochondrial levels of mtDNA enzymes are dependent on mtROS signaling.

### MtROS induce mitochondrial functional deficiency and structural impairment in the heart after sepsis

**Fig 3:**
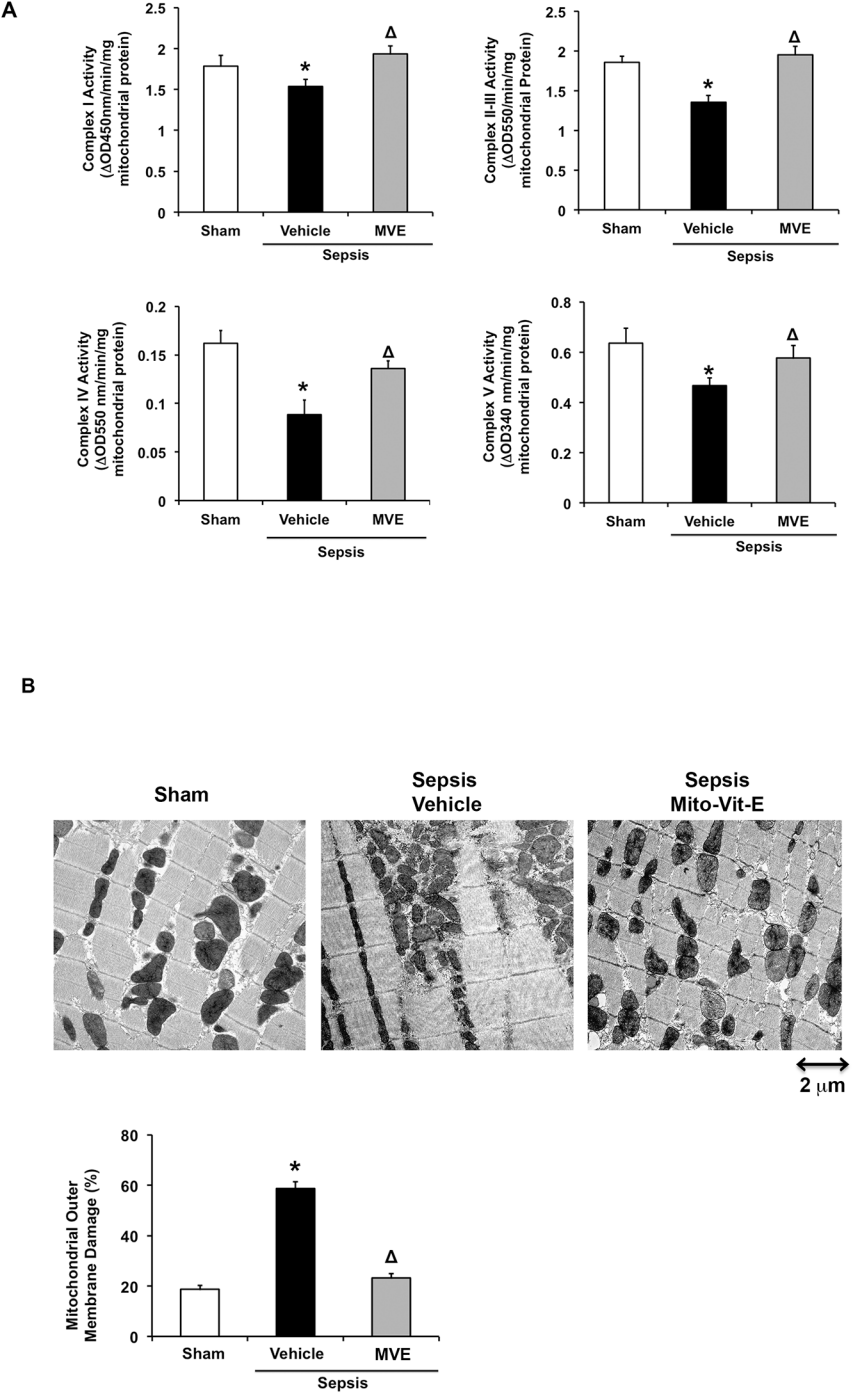
Mitochondrial ROS dependent functional deficiency and structural impairment in cardiac mitochondria after sepsis. Rats were infected by *S*. *pneumoniae* or given PBS sham control. 21.5 μmoles/kg Mito-Vit-E (MVE) or vehicle was administered orally 30 minutes post-inoculation, and heart tissues were harvested 24 hours later. **A.** Mitochondrial fractions were subjected to the measurements of complex I-V activities. **B.** Ultrastructure of myocardial mitochondria was observed by transmission electron microscope (TEM). Biochemically, mitochondrial outer membrane damage was measured using the mitochondrial fractions. All values are means ±SE. Significant differences are shown as * between sham and sepsis and Δ between vehicle and Mito-Vit-E (*p*<0.02, n = 6).

Since mtDNA encodes 37 genes that are essential for normal mitochondrial function [57], mitochondrial metabolism and structure are expected to alter in response to mtDNA damage. We thus measured the activities of mitochondrial oxidative phosphorylation (OXPHOS) complexes in the hearts of sham and septic rats given Mito-Vit-E or control vehicle. We found that the activities of complex I, II-III, IV and V were significantly reduced in response to sepsis, decreased ~20%, ~30%, ~60% and ~25% respectively (Fig 3A). The decrease in the activity of the complex V, ATP synthase, directly indicates that the capacity of energy production in the heart is compromised during sepsis. Intriguingly, these deficiencies were recovered by Mito-Vit-E, suggesting it is sufficient to provide protection for mitochondrial function.

In addition, we examined mitochondrial structural integrity in the heart tissue morphologically and biochemically. As shown in Fig 3B, ultrastructual observation under electron microscopy (EM) indicates that sepsis induced an apparent mitochondrial membrane disruption in cardiomyocytes, and it was alleviated by Mito-Vit-E. Visible electron dense particles in the septic subjects are precipitation of glycogen, which level increases under stress conditions [58]. Additionally, we estimated mitochondrial membrane integrity by the measurement of cytochrome C oxidase (COX) activity in mitochondrial fractions in the presence and absence of detergent n-dodecyl β-D-maltoside [59]. Because COX is located on the inner mitochondrial membrane, the comparison between its activities without and with the detergent is a relative ratio of intact and total mitochondria, representing mitochondrial outer membrane integrity [53]. Consistent with our previous report [47], sepsis triggered a two-fold increase in the damage of mitochondrial membrane, whereas Mito-Vit-E provided an effective protection under this pathological condition.

### MtROS-dependent activation of mtDNA-TLR9-RAGE pathway in the heart after sepsis

**Fig 4:**
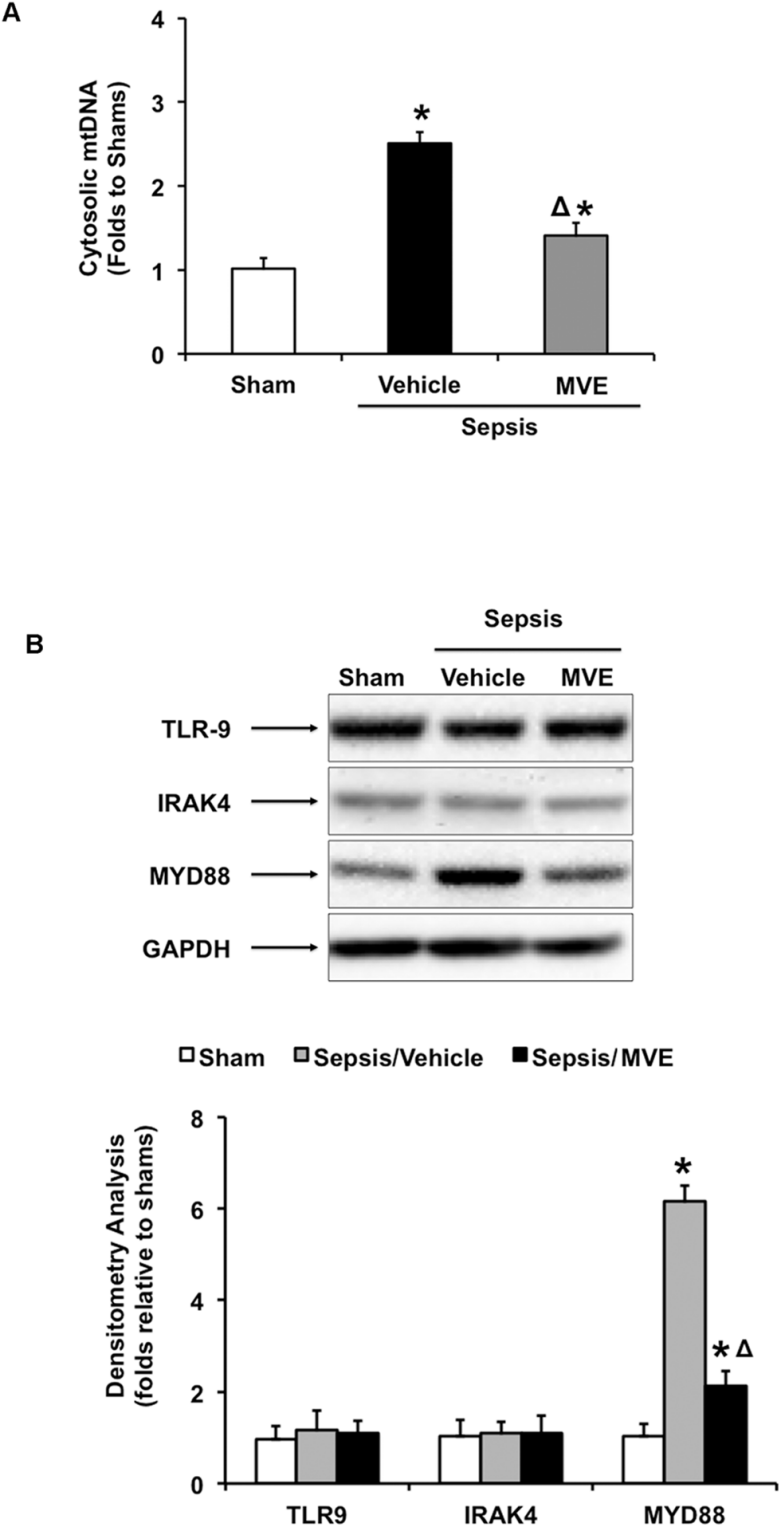
Mitochondrial ROS dependent activation of mtDNA-TLR9 pathway in the heart after sepsis. Rats were infected by *S*. *pneumoniae* or given PBS sham control. 21.5 μmoles/kg Mito-Vit-E (MVE) or vehicle was administered orally 30 minutes post-inoculation, and heart tissues were harvested 24 hours later. **A**. Levels of mtDNA in cytosol fractions were measured by real-time PCR. **B**. Expression of TLR9, IRAK4 and MyD88 were determined in total tissue lysates by western blot using GAPDH as a loading control, and the results were quantified by densitometry. All values are means ±SE. Significant differences are shown as * between sham and sepsis and Δ between vehicle and Mito-Vit-E (*p*<0.02, n = 6).

Damage in mtDNA often leads to mtDNA fragmentation, and mtDNA fragments released from injured mitochondria circulate as danger-associated molecular patterns (DAMPs) to induce downstream inflammatory responses [11, 27]. By RPCR analysis, we compared levels of mtDNA leaked to cytoplasm in the heart of sham and septic rats given Mito-Vit-E or control vehicle. As summarized in Fig 4A, RPCR targeting a mtDNA-specific gene sequence of NADH dehydrogenase detected a ~1.5-fold-increase of mtDNA that were released into the myocardial cytoplasm in response to sepsis. In septic rats receiving Mito-Vit-E, the level of cytosolic mtDNA was significantly reduced, yet still remained slightly higher, still showing a statistical significance when compared to sham control animals. This result suggests that specific inhibition of mtROS limits the accumulation of cytosolic mtDNA, a type of DAMP molecules, in the heart after sepsis.

TLR9 is essential for the recognition of mtDNA fragments, which are rich in unmethylated CpG motif and similar to bacterial DNA [11, 29, 30]. We next examined whether sepsis alters the expression of TLR9 and related response factors, such as myeloid differentiation primary response protein MYD88 [60, 61] and IL-1R-associated kinase–4 (IRAK4) [62]. We also examined whether intervention of Mito-Vit-E affected any of these changes. As shown in Fig 4B, Western blot analysis detected a ~5-fold increase in MYD88 expression in septic subjects, compared with their sham counter parts. However, no significant changes were detected in the expression of TLR9 and IRAK4. Further, sepsis-induced expression of MYD88 was suppressed in animals treated with Mito-Vit-E, suggesting a mtROS-dependent signaling response.

**Fig 5:**
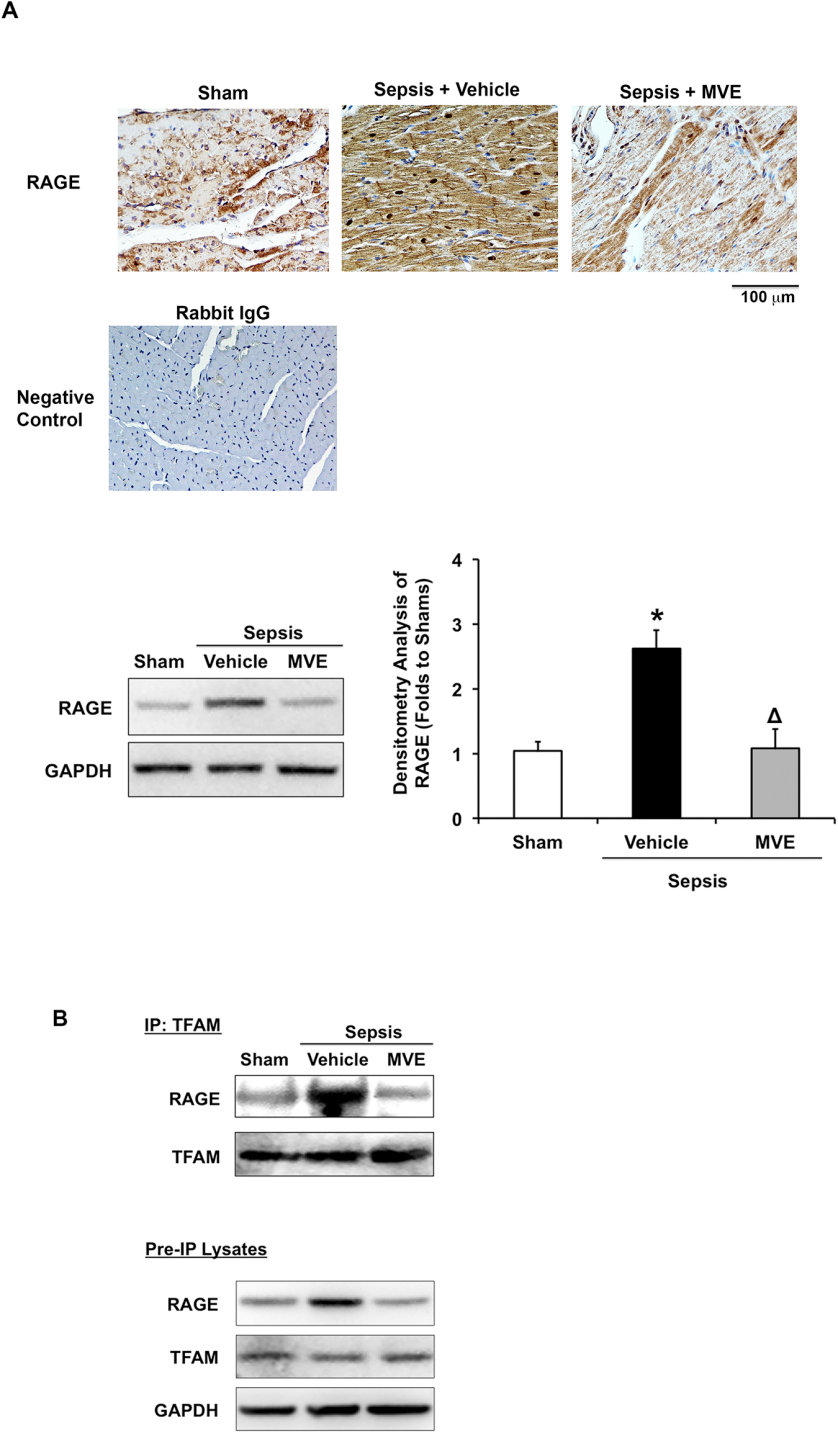
Mitochondrial ROS dependent activation of RAGE pathway in the heart after sepsis. Rats were infected by *S*. *pneumoniae* or given PBS sham control. 21.5 μmoles/kg Mito-Vit-E (MVE) or vehicle was administered orally 30 minutes post-inoculation, and heart tissues were harvested 24 hours later. **A**. Heart sections were stained with anti-RAGE (brown) and haematoxylin (blue). Negative control was stained with secondary antibody alone. Images are representative of a random selection of at least 3 sections of N = 6. **B**. RAGE-TFAM interaction was determined by co-immunoprecipitation in total heart lysate. Shown result was reprehensive of three independent experiments (N = 6).

Recent investigations implicate RAGE and TFAM in the regulation of inflammatory responses to circulating DNA [31, 33, 34, 63]. RAGE expresses in multiple tissues and its up-regulation in cardiac tissue were found associated with heart failure [64, 65]. We applied both immunostaining and western blot analysis to examine the expression pattern of RAGE in the heart of sham and septic rats receiving Mito-Vit-E or control vehicle. As shown in Fig 5A, we detected a significant, over 2.5-fold increase in RAGE expression in response to sepsis, and this increase was effectively inhibited by Mito-Vit-E. From the result of immunostaining, it was noticed that Mito-Vit-E had a stronger inhibitory effect on RAGE expression in cardiomyocytes than it did in inflammatory cells in myocardium. The difference maybe due to the fact that mitochondria-enriched cells such as cardiomyocytes accumulate more mtROS. Since RAGE signaling is mediated by mtROS, cardiomyocytes react to the treatment by Mito-Vit-E more quickly and significantly in response to sepsis.

RAGE functions as a receptor for multiple ligands including the high-mobility group box 1 protein HMGB1 and mitochondrial transcription factor TFAM [31–33]. We next examined if RAGE is physically associated with TFAM and/or HMGB1 in septic hearts and whether this association is dependent on mtROS signaling. As shown in Fig 5B, results from co-immunoprecipitation detection suggested a strong interaction between RAGE and TFAM in septic animals. This RAGE-TFAM interaction was modest either in shams or in septic rats treated with Mito-Vit-E. Considering the subcellular location of TFAM in mitochondria but RAGE on membrane or in cytosol [66], we suspect that the RAGE-TFAM interaction observed here is a result of mtROS-triggered increases in mitochondrial damage, leading to the leakage of TFAM to the cytoplasm. This reaction is aggravated by the overwhelming expression of RAGE after sepsis (shown in Fig 5A and in pre-IP lysates in Fig 5B).

### MtROS-dependent activation of inflammatory pathways in the heart after sepsis

**Fig 6:**
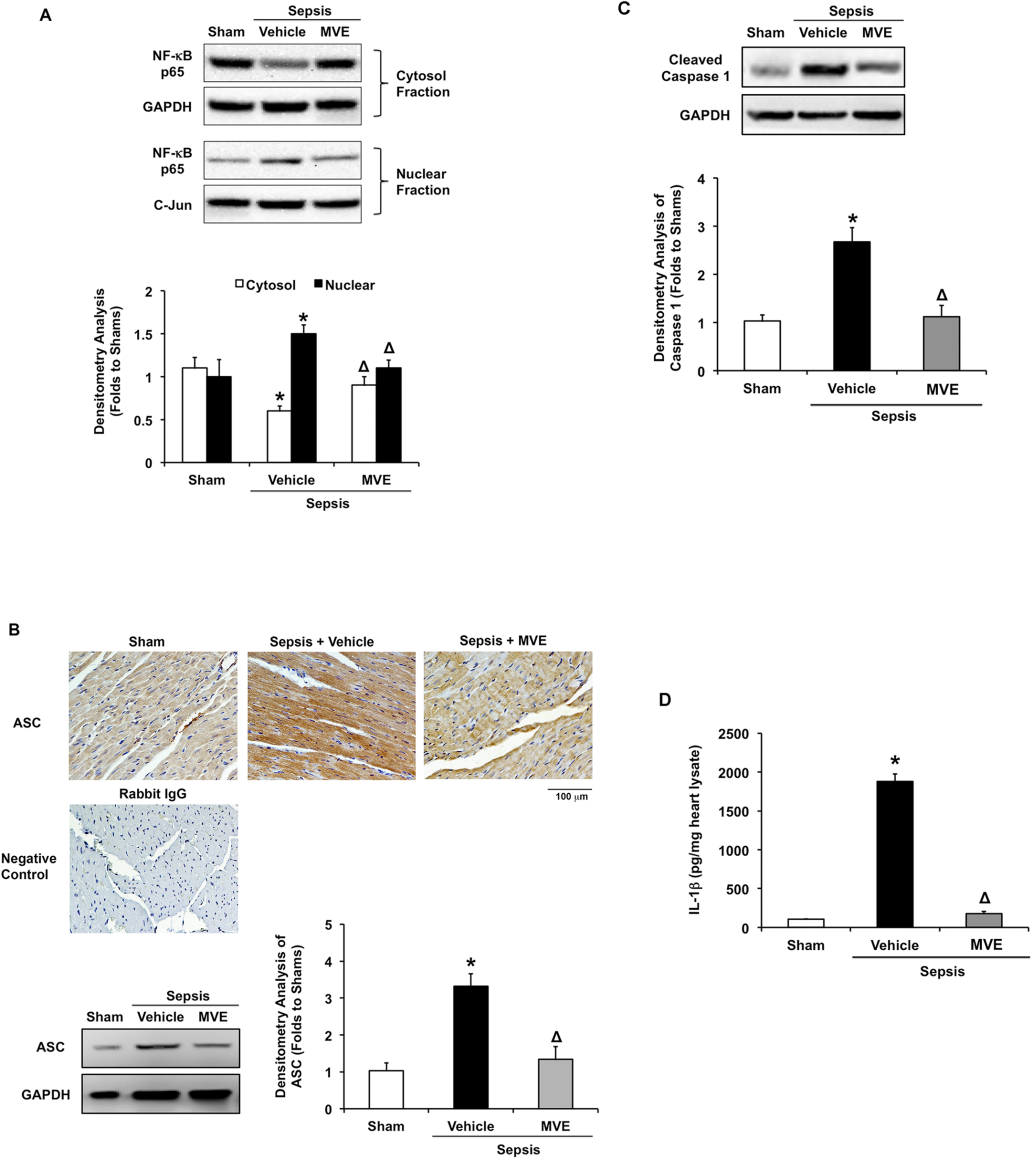
Mitochondrial ROS-dependent activation of myocardial inflammation after sepsis. Rats were infected by *S*. *pneumoniae* or given PBS sham control. 21.5 μmoles/kg Mito-Vit-E (MVE) or vehicle was administered orally 30 minutes post-inoculation, and heart tissues were harvested 24 hours later. **A.** NF-κB p65 subunit was detected in cytosolic and nuclear fractions by Western blot using GAPDH and c-Jun as a loading control respectively. Results were quantified by densitometry. **B.** Heart sections were co-stained with anti-ASC (brown) and haematoxylin (blue). Negative control was stained with secondary antibody alone. Images are representative of a random selection of at least 3 sections of N = 6. **C.** Activated form of caspase 1 was determined in heart tissue lysates by Western blot using GAPDH as a loading control, and results were quantified by densitometry. **D.** Production of IL–1β was measured in heart tissue lysates by ELISA. All values are means ±SE, and statistical significances are shown as * between sham and sepsis and Δ between vehicle and Mito-Vit-E (*p*<0.02, n = 6).

We previously showed that treatment with Mito-Vit-E suppressed cytokine levels in heart tissue in the pneumonia-related sepsis model [47]. To study the mechanism underlying the anti-inflammatory effects of Mito-Vit-E, we examined whether Mito-Vit-E affects the activation of NF-κB. Activation of NF-κB is associated with its translocation from the cytosol to the nucleus. Consistent with previous observation [46], the p65 subunit of NF-κB was significantly decreased in the cytosol but increased in the nucleus in the heart of septic animals, compared with sham controls (Fig 6A). This NF-κB translocation was not detected in rats given Mito-Vit-E, suggesting a regulatory role of mtROS in the activation of NF-κB pathway in septic hearts.

Inflammasomes exert critical transcriptional regulation of certain cytokines such as IL–1β[67, 68]. We examined the effects of sepsis and Mito-Vit-E on the expression of inflammasome adaptor protein ASC (apoptosis-associated speck-like protein containing a C-terminal caspase recruitment domain) in the heart. As shown in Fig 6B, we observed a significant increase in myocardial ASC expression in septic rats, compared with shams, by immunohistochemistry analysis. This increase was estimated about 2.5-fold by Western blot. Results obtained from both methods revealed that giving Mito-Vit-E suppressed this sepsis-induced up-regulation of ASC. Similar to what we observed in RAGE expression (Fig 5A), Mito-Vit-E provided stronger inhibitory effect on ASC expression in cardiomyocytes than it did in inflammatory cells, which is presumably due to the fact that cardiomyocytes are high in mitochondria density and mtROS concentration, thus significantly respond to Mito-Vit-E in a timely fashion after sepsis. Furthermore, we examined caspase 1 activation and IL–1β production, as both are down-stream events following ASC inflammasome formation. We detected that sepsis stimulates an over 2-fold increase in accumulation of the active form of caspase 1, resulting in a nearly 2000-fold induction of IL–1β generation, and Mito-Vit-E profoundly suppressed both events (Fig 6C and 6D).

Together, these data suggest that sepsis induces mtROS-dependent inflammatory responses in the heart, and that mitochondria-targeted antioxidants may provide an effective control over this pathologic response after sepsis.

### MtROS-dependent inflammation in cardiomyocytes in response to LPS

**Fig 7:**
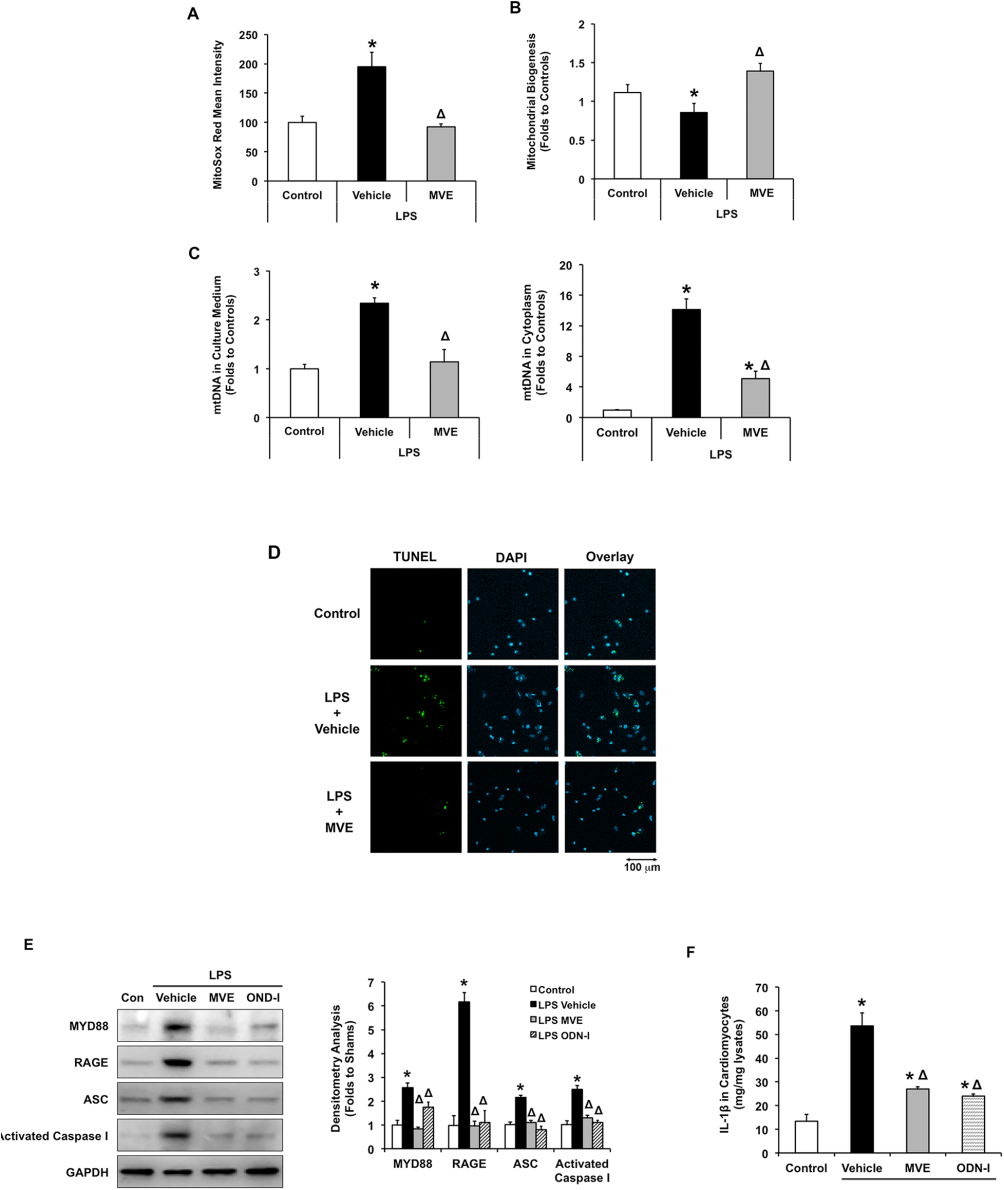
Effects of Mito-Vit-E and TLR9 inhibitor OND-I in LPS-challenged cardiomyocytes. Cultured neonatal cardiomyocytes from rats were treated with ±LPS (100 ng/ml), ±Mito-Vit-E (MVE) (1μM), or ±ODN-I (0.5 μM) 4 hours prior to harvesting. **A.** Mitochondrial superoxide was labeled with MitoSox Red and quantified by flow cytometry. **B**. Mitochondrial biogenesis was quantified in live cells using MitoBiogenesis In-Cell ELISA assay. **C.** Levels of mtDNA in cell medium and in cytoplasm were measured by real-time PCR. **D**. Cells apoptosis was evaluated by TUNEL assay (green). Cell nucleuses were identified by DAPI staining (blue). **E.** Expression of MyD88, RAGE, ASC and activated form of caspase 1 were determined in cell lysates by western blot using GAPDH as a loading control, and results were quantified by densitometry. **F.** Cellular production of IL–1β was measured by ELISA. All the measurements were normalized by cell numbers and obtained in triplicate. All values are means ±SE. Significant differences are shown as * between control and LPS and Δ between vehicle and drug-treated groups (*p*<0.02 for A-C and p<0.01 for E-F, n = 4).

Heart tissue is composed of multiple cell types. To validate whether sepsis induces mtROS-dependent cardiac inflammation occurs directly in cardiomyocytes, we examined the response of cultured primary neonatal cardiomyocytes under the condition of LPS-challenge. As expected, in these cells, Mito-Vit-E effectively suppressed mtROS production, detected by MitoSox Red, a florescent dye specifically targeted at mitochondrial superoxide (Fig 7A). Mito-Vit-E also significantly ameliorated LPS-triggered deficiency in mitochondrial biogenesis (Fig 7B), and decreased the release of mtDNA fragments to extracellular space. In addition, though not completely, Mito-Vit-E reduced a drastic, over 65% of the mtDNA release in cytoplasm in LPS-challenged cells (Fig 7C). We hypothesize that these mtDNA fragments were freed from damaged mitochondria and dead cells. Indeed, under these conditions, LPS stimulated apoptosis and this response was attenuated by Mito-Vit-E (**Fig 7D**). Both Mito-Vit-E and OND-I (ODN 2088), a TLR9 antagonist, provided significant inhibition on LPS-induced expression of TLR9 pathway factors MYD88 and RAGE. ASC expression was also inhibited, and so were its down stream activation of caspase 1 and production of IL–1β (Fig 7E and 7F). These results obtained *in vitro* are consistent with the *in vivo* findings described above, supporting the notion that sepsis signals an inflammatory pathway mediated through mtROS-mtDNA-TLR9-RAGE in the heart.

### Targeted inhibition of mtROS ameliorates sepsis-induced cardiac damage

In our previous evaluation of Mito-Vit-E in the sepsis model, measurement of the cardiac shortening fraction indicated that this compound improved cardiac performance [47]. The anti-inflammatory effects of Mito-Vit-E were implicated by a significant reduction in cytokines and an inhibition of neutrophil accumulation in the myocardium, signified by the suppression of the quantity and activity of neutrophil marker myeloperoxidase [47]. In this report, we further evaluated whether Mito-Vit-E reduces myocardial damage after sepsis using the measurement of serum troponin-I (cTn-I) and the analysis of cardiac histopathology.

**Fig 8:**
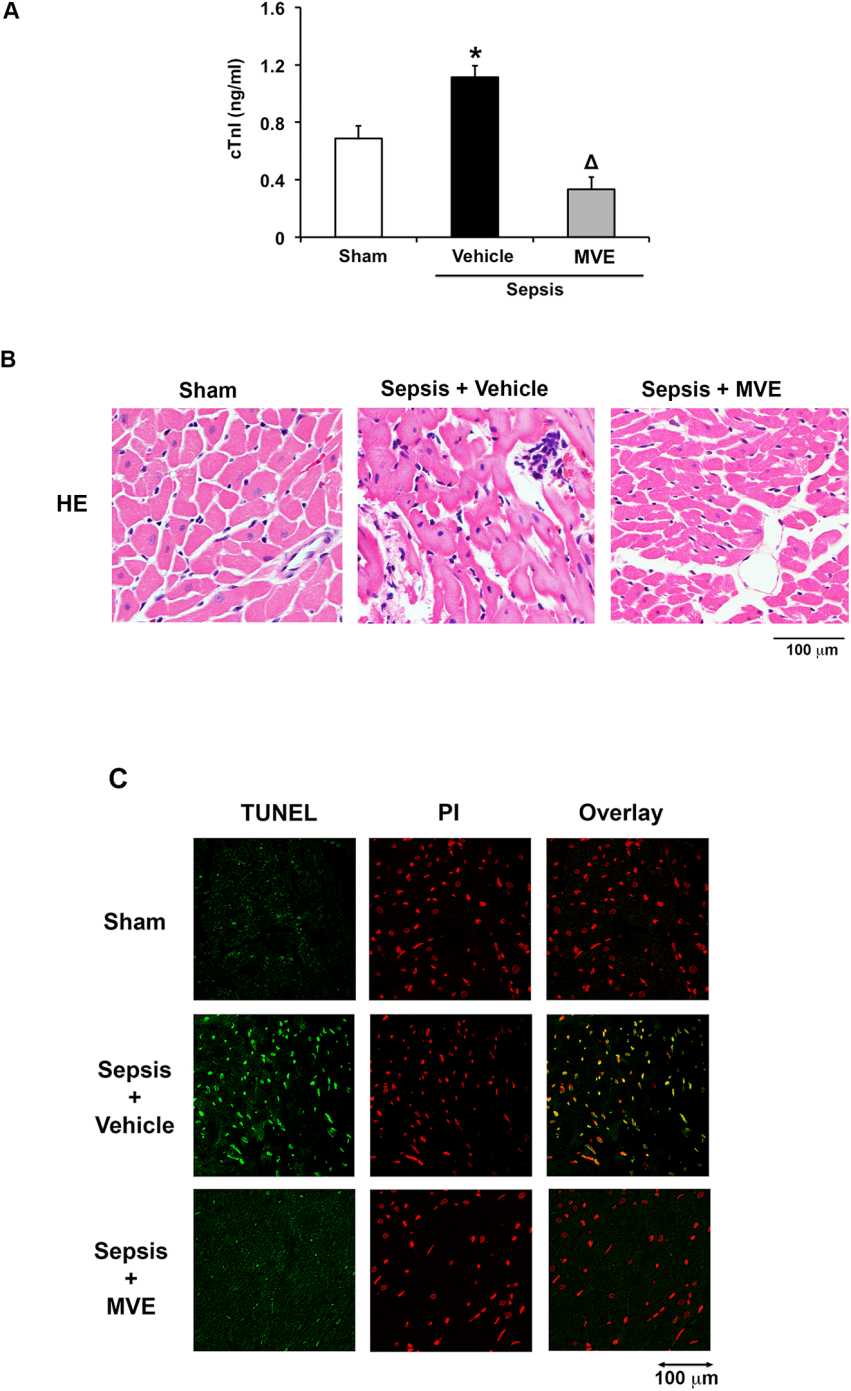
Mito-Vit-E attenuates myocardial damage after sepsis. Rats were infected by *S*. *pneumoniae* or given PBS sham control. 21.5 μmoles/kg Mito-Vit-E (MVE) or vehicle was administered orally 30 minutes post-inoculation, and heart tissues were harvested 24 hours later. **A**. Serum levels of troponin I (cTnI) were measured by ELISA assay. All values are means ±SE. Significant differences are shown as * between sham and sepsis and Δ between vehicle and Mito-Vit-E (*p*<0.05, n = 6). In addition, heart tissue sections were applied to H&E staining (**B)** and TUNEL assay (green) (**C**). In **C**, cell nucleuses were identified by propidium iodide (PI) staining (Red). The original magnification is 40 fold, and images are representative of a random selection of at least 3 sections of N = 6.

Clinically, sepsis results in a substantial elevation in cTn-I [69], one of the most sensitive indicators of myocardial injury [70]. We compared the levels of serum cTn-I in the sham and septic rats given with Mito-Vit-E or control vehicle. As shown in Fig 8A, cTn-I increased significantly in septic rats compared with sham animals, and Mito-Vit-E effectively suppressed this response. In addition, we performed histological analysis of the heart tissue by hematoxylin and eosin staining (H&E stain). As expected, sepsis caused significant accumulation of blood cells in myocardium and disorganization of myocardium (Fig 8B). These changes were not detected in septic rats receiving Mito-Vit-E. Mito-Vit-E’s effect on myocardial apoptosis after sepsis was also evaluated by terminal deoxynucleotidyl transferase dUTP nick end labeling (TUNEL) to detect DNA fragmentation. As shown in Fig 8C, sepsis stimulated a striking increase in DNA fragmentation in the heart tissue, and giving Mito-Vit-E in septic rats provided an almost complete attenuation of this response. Taken together, the data indicate that targeted suppression of mtROS ameliorates sepsis-induced myocardial damage.

## Discussion

**Fig 9:**
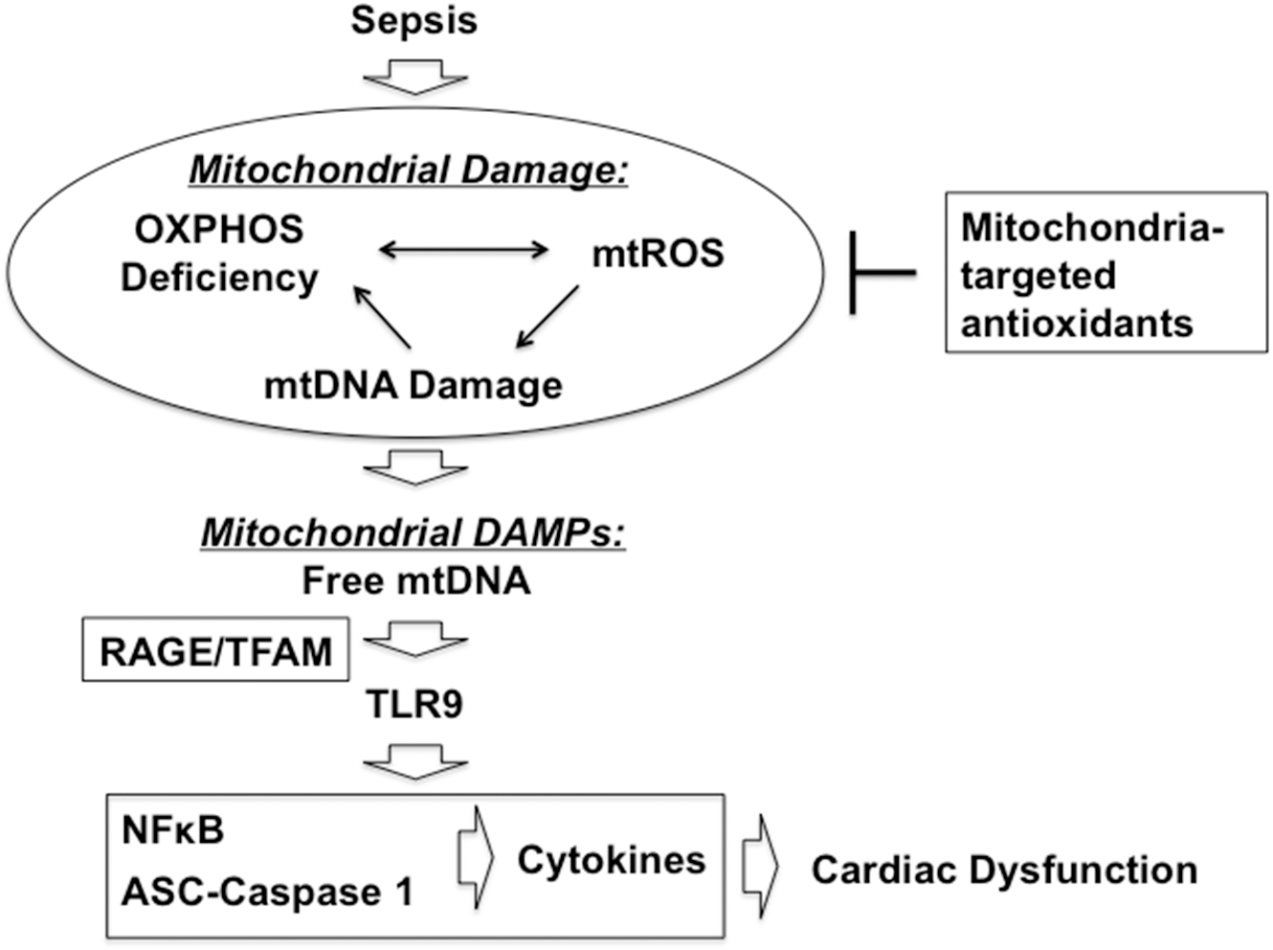
Mitochondrial ROS-induced cardiac damage during sepsis and the mechanism of action of mitochondria-targeted antioxidants (MTAs).

We previously evaluated mitochondria-targeted vitamin E (Mito-Vit-E) in a pneumonia-related rat sepsis model and obtained evidence showing that this compound specifically inhibits mtROS, suppresses cardiac inflammation and improves cardiac function after sepsis [47]. In this report, using the same mitochondria-targeting strategy in the same model, we investigated the signaling pathway(s) underlying mtROS-mediated inflammation in the heart after sepsis. Compared with conventional vitamin E, Mito-Vit-E provided significantly diminished mtDNA damage in the heart, indicated by its restoration of mtDNA integrity and reduction in mtDNA oxidative modification (Fig 1). In addition, Mito-Vit-E improved levels of DNA repair enzymes within the mitochondria (Fig 2). Mito-Vit-E’s mitochondrial protection was further supported by its attenuation both in metabolic deficiency and in impairment of membrane structure in the septic heart (Fig 3). As a consequence of mitochondrial damage after sepsis, cytosolic free mtDNA fragments, potentially functioning as DAMPs to incite inflammation via TLR9 [71], were significantly increased in the heart (Fig 4A). Supporting the hypothesized role of TLR9 pathway in sepsis, our results showed an increase in myocardial expression of MYD88 and RAGE, both of which function as downstream TLR9 factors, as well as a formation of physical interaction between RAGE and its ligand TFAM (Figs 4B and 5). Treatment with Mito-Vit-E attenuated these changes, implicating mtROS as critical causative factors to stimulating inflammatory responses. We also provide data demonstrating that the mechanism of Mito-Vit-E’s anti-inflammatory action involved the down-regulation of NF-κB activation, expression of inflammasome ASC and production of inflammatory cytokines such as IL–1β (Fig 6). Similar to the *in vivo* findings, Mito-Vit-E suppressed mtROS, improved mitochondrial function, reduced free extracellular mtDNA and prevented apoptosis *in vitro* in LPS-challenged neonatal cardiomyocytes. Both Mito-Vit-E and TLR9 inhibitor OND-I repressed LPS-induced up-regulation of MYD88, RAGE, ASC, activated caspase 1 and IL–1β, confirming that sepsis induces a mtROS dependent mtDNA-TLR9 pathway in cardiomyocytes (Fig 7). We previously showed that Mito-Vit-E improved cardiac performance in septic rats [47]. This study obtained new supporting evidence clearly showing its reduction in cardiac injury marker troponin-I, decrease in apoptosis and amelioration of cardiac morphology (Fig 8). Based on these results, we propose that sepsis-induced mtROS damage mtDNA, thus then leads to the release of free mtDNA and the activation of a TLR9 pathway, which is, at least in part, responsible for the development of cardiac failure after sepsis. Targeting mtROS by mitochondria-targeted antioxidants presents a promising therapeutic potential to provide cardiac protection in sepsis (Fig 9).

Abnormalities of cardiac mtDNA have been found to associate with a number of heart problems, such as ischemic heart disease [72] dilated cardiomyopathy [73], cardiac hypertrophy [74], and heart failure after myocardial infarction [75]. Though it remains unclear how sepsis stimulates mtDNA damage in myocardium, our data suggest that cardiac mtDNA damage is a direct result of mtROS overproduction after sepsis. In the heart of septic rats, we first observed a decrease in antioxidant activities in mitochondria [46]. Secondarily, we identified that altered mitochondria-localization of tyrosine kinase Src and phosphatase SHP2 causes a significant down-regulation of tyrosine phosphorylation in certain mitochondrial structural and functional proteins, including subunits of respiratory complex I and III, and thus leads to deficient mitochondrial function [48]. Since a large portion of mtROS are generated from reactions of complex I and III [76], deregulated mitochondrial localization of Src and SHP2 potentially constitutes a critical signaling pathway to accumulate mtROS and thus causes massive oxidative damage in the heart during sepsis. In the present study, we detected an approximately 30% reduction in the content of intact mtDNA in the heart of septic rats (Fig 1A). This response appeared driven by mtROS, since mtDNA content was effectively protected by Mito-Vit-E that suppresses mtROS specifically [47]. Consistently, sepsis triggers the accumulation of oxidative damage in mtDNA, marked by 8-OH-dG lesions and abasic sites, and as expected, this damage was attenuated by Mito-Vit-E intervention (Fig 1B and 1C).

Reported observations have suggested that mtDNA repair enzymes, such as mitochondria-specific polymerase γ (POLG), might become targets of oxidative damage [77]. In this report, we found that mitochondria-located levels of POLG and mtDNA base excision proteins including apurinic/apyrimidinic (AP)-endonuclease, 8-oxoguanine glycosylase (OGG1) and uracil-DNA glycosylase (UNG1) were significantly reduced in septic animals but not in Mito-Vit-E treated subjects (Fig 2). This result suggests that sepsis may cause mtROS-dependent changes in mtDNA enzymes in the heart, such as disturbance in gene expression, increase in oxidative modification and/or impairment of mitochondrial transportation. These possibilities will be further investigated in our future studies. Taken together, our current data suggest that sepsis-induced mtROS cause mtDNA damage directly via oxidative modification and indirectly via impairment of mtDNA repair function in the heart. Since mtDNA encodes 37 genes that are essential for normal mitochondrial function [57], mitochondrial respiratory activities and mitochondrial structure were severely altered in septic hearts, and Mito-Vit-E provided an effective control to alleviate sepsis-induced mitochondrial functional deficiency and structural damage **(Fig 3).**

In addition to be the intracellular powerhouse and the key regulators of cell death, mitochondria are a critical source of danger-associated molecular patterns (DAMPs) to stimulate inflammation [11]. In response to tissue injuries, deficiencies in mitochondrial function and impairment in mtDNA result in the release of free mtDNA fragments into the cytoplasm from raptured mitochondria and later to extracellular spaces from dead cells. These mtDNA vary in sizes but are recognized by TLR9 through unmethylated CpG motif, thus function as DAMPs to induce downstream inflammation via a TLR9 pathway [11, 27, 29, 30, 47]. In the hearts of septic animals, we detected that cytosolic mtDNA was increased over 100% (Fig 4A), in association with a significant up-regulation of MYD88, a common adaptor protein recruited by TLRs including TLR9 [60, 61] (Fig 4B). On the other hand, the myocardial expression of TLR9 itself and IRAK4, a factor downstream of TLR-MYD88, was not affected in the sepsis model we examined. In this study, the link between mtROS-mediated mtDNA damage and activation of the TLR9 pathway is further indicated by the increases in RAGE expression and RAGE-TFAM interaction (Fig 5). Recent research suggests that TFAM, a structural homolog of HMGB1 [32] and a ligand of RAGE [33], utilizes RAGE to promote cytokine production through a cytosolic DNA-TLR9 dependent signaling pathway [31, 34]. We found that sepsis stimulates staggering increases in both RAGE expression and its physical interaction with TFAM, and that both responses are mtROS-dependent. We therefore hypothesize that, in septic heart, mtROS-damaged mitochondria release mtDNA fragments and TFAM into the cytoplasm, which, in turn, incites a TLR9-mediated inflammatory responses via RAGE. These results are consistent with the conclusion that TFAM functions as one of the DAMPs during inflammation [31, 34]. In our experimental settings, we detected neither an alteration in HMGB1 expression nor an interaction between HMGB1 and RAGE (data not shown). Thus, although both HMGB1 and TFAM possess ligand-binding activities for RAGE, these factors may respond to tissue- or cell-specific signaling in various pathological conditions. Additionally, it is also noteworthy that other TLRs, such as TLR2 and TLR4, present pivotal roles in the regulation of sepsis-induced cardiac inflammation during sepsis [78, 79]. Future investigation of how cardiac inflammation is regulated via different TLR pathways, spatially and temporally, or by their crosstalk is important to understand the pathological mechanism underlying sepsis-induced cardiac dysfunction.

In blood, systemic inflammatory responses are activated through IKK signalosome pathway, in which NF-κB is negatively regulated by the IκB kinase (IKK) signalosome pathway [80, 81], and through inflammasome pathways, in which the inflammatory caspases 1 and 5 are controlled by the inflammasomes [82–84]. This signal transduction paradigm is very likely applicable to the tissue-level inflammation observed during sepsis. Indeed, inflammatory responses in various organs, such as lung, liver, kidney, heart, *etc*. have been intensively studied in sepsis models [85–89]. In the pneumonia-related sepsis model, we previously identified NF-κB activation and cytokine overproduction in the heart [46]. We further showed that treatment with Mito-Vit-E suppressed myocardial cytokines in this model [47]. Results presented here showed that Mito-Vit-E inhibited the activation of both NF-κB and ASC-regulated inflammasome pathways (Fig 6). Thus, our investigation suggests that sepsis-induced cardiac inflammation is mtROS-dependent, presumably due to the drastic release of mitochondrial DAMPs.

We performed similar investigations *ex vivo* in LPS-challenged cardiomyocytes. Our data indicate that Mito-Vit-E targeted suppressed mtROS in these cells (Fig 7), consistent with previous evaluation of this group of antioxidants in various disease models by our lab and others [47, 90–93]. LPS triggered increases in mtDNA release, apoptotic response, expression of inflammatory mediators including MYD88, RAGE, ASC, IL–1β and activation of caspase 1. Inhibition of these responses by both Mito-Vit-E and TLR9 inhibitor ODN-I provides clear support of our hypothesis that sepsis stimulates a mtROS-mtDNA-TLR9 inflammation pathway in cardiomyocytes.

Currently, novel mitochondria-targeted antioxidants (MTAs) have demonstrated, in various experimental settings, their ability to ablate oxidative stress and to protect mitochondrial function [90–93]. The therapeutic potential of this group of molecules has been under intensive investigation using pre-clinical models of mitochondrial abnormalities-associated diseases such as neurodegenerative diseases [94, 95], cardiac dysfunction [96], cardiac ischemia-reperfusion injury [97], hypertension [98], diabetes [99], and sepsis [100, 101]. Current investigations evaluated MitoQ in septic animal models and the available data suggest that it has significant therapeutic benefits in improving cardiac function in endotoxin-induced sepsis [101] and in preventing liver damage in lipopolysaccharide-induced sepsis [100]. Our recent testing of Mito-Vit-E in pneumonia-related sepsis model obtained data showing that it suppressed cytokines and improved cardiac contractility in sepsis [47]. Studies described in this report revealed the possible mechanism underlying Mito-Vit-E’s anti-inflammatory action. We also showed that this compound reduced circulating troponin I, improved tissue morphology, and attenuated apoptosis in the heart of septic rats (Fig 8). Clinically, elevated cardiac risk factor troponin levels reflect higher disease severity, myocardial dysfunction and worse prognosis in sepsis patients [102]. Mito-Vit-E’s cardiac protective effects are presumably delivered through its inhibition on mtROS-mediated pathological responses such as apoptosis and inflammation. In addition, the signaling pathways observed here, such as production of mtDNA DAMPs, activation of NF-κB, RAGE and ASC, incite inflammation not only in the heart but also in other organs [103–107]. Thus, Mito-Vit-E, as well as other MTAs, potentially provides an effective therapeutic approach to limit multi-organ failure after sepsis. Further studies of MTAs in different sepsis models and different organs will promote translating the application of these novel molecules into significantly improved clinical outcomes.
